# Modeling of Current Consumption in 802.15.4/ZigBee Sensor Motes

**DOI:** 10.3390/s100605443

**Published:** 2010-06-01

**Authors:** Eduardo Casilari, Jose M. Cano-García, Gonzalo Campos-Garrido

**Affiliations:** Departamento Tecnología Electronica, University of Malaga, ETSIT, Campus de Teatinos, 29071 Malaga, Spain; E-Mails: cano@dte.uma.es (J.M.C-G); gnz83@hotmail.com (G.C-G)

**Keywords:** 802.15.4/ZigBee, battery, sensor network

## Abstract

Battery consumption is a key aspect in the performance of wireless sensor networks. One of the most promising technologies for this type of networks is 802.15.4/ZigBee. This paper presents an empirical characterization of battery consumption in commercial 802.15.4/ZigBee motes. This characterization is based on the measurement of the current that is drained from the power source under different 802.15.4 communication operations. The measurements permit the definition of an analytical model to predict the maximum, minimum and mean expected battery lifetime of a sensor networking application as a function of the sensor duty cycle and the size of the sensed data.

## 1. Introduction

The IEEE 802.15.4 standard (which describes the Physical Layer and Medium Access Control [1]) and ZigBee [2] jointly specify a protocol stack for the development of short-range and low power communications for Wireless Personal Area Networks (WPANs). This stack is aimed at providing networking architectures for low-cost wireless embedded devices with consumption and bandwidth limitations. In particular the basic framework of IEEE 802.15.4 permits up to 10-meter communications with a transfer rate of 250 kbps, although this parameter can be decreased even more (down to 20 Kbps in the 868/915 MHz band) to enable a lower power consumption in the ZigBee nodes. IEEE 802.15.4-compliant transceivers, which operate in the Industrial, Scientific and Medical (ISM) radio bands (including 2.4 GHz band), are designed to be simpler and more economical than the modules from other candidate standards for WPANs (such as Bluetooth). Additionally, the standard also contemplates the possibility of real-time guarantees so it can be also put into operation in scenarios where real-time flows (e.g., voice or the signal from a biosensor such as an electrocardiogram) are expected to be transmitted. As a consequence, in spite of the immature state of ZigBee technology, it has become an appealing alternative to support a wide set of services, particularly for low consume domotic sensor networks but also for other applications ranging from medical telemonitoring to industrial plant-process control.

The main attractiveness and also the main challenge of IEEE 802.15.4/ZigBee is its potentiality to set up self-organizing networks capable of adapting to diverse topologies, node connectivity and traffic conditions. Typical applications of 802.15.4/ZigBee usually consist of tens or hundreds of simple battery-powered sensor nodes (or “motes”) which periodically (or sporadically) transmit their sensed data to one or several data sinks (acting as the ZigBee Coordinator or intermediate routers in the way to the Coordinator).

ZigBee/802.15.4 technology was conceived to minimize the power consumption of these motes. For this purpose, the activity of the motes must be reduced up to a minimum so they can remain most of the time in a low-power (sleep) state. Therefore, a mote just has to ‘wake up’ (to be active) in order to sense and transmit (or receive) the data for a small fraction of time. The general objective is to maximize the lifetime of the battery in the motes and consequently the lifetime of the sensor network itself.

In order to predict the battery duration of the devices in a practical implementation of a 802.15.4/ZigBee network, we must characterize the activity cycles of the motes as well as the current which is drained from the battery during the different operations imposed by the dynamics of 802.15.4 communications, especially those which imply the activation of the radio transceiver (main source of battery consumption in the motes).

In the literature there are studies that model the power consumed by devices which utilize proprietary stacks or just the physical layer of 802.15.4 (see, for example, the study in [3] about the CC1000 radio module of Mica2 motes by Crossbow [4]). In this paper we present an experimental testbed developed to empirically model the power consumption of commercial motes that implement the entire 802.15.4/ZigBee stack. The obtained results are applied to define a model which, in different circumstances, permits to forecast the battery lifetime of the motes in a wireless sensor network.

This paper is organized as it follows: Section 2 outlines the main characteristics of 802.15.4 Medium Access Control. Section 3 reviews the existing literature on the characterization of 802.15.4/ZigBee. Section 4 describes the experimental testbed, while Section 5 presents and comments the performed measurements. The model for battery lifetime is explained in Section 6. Finally, Section 7 summarizes the main conclusions of the paper.

## 2. Overview of 802.15.4 Medium Access Control

Many possible advantages of employing IEEE 802.15.4 are strongly determined by the configuration of the Medium Access Control (MAC) sublayer. In this sense the IEEE 802.15.4 standard distinguishes two classes of nodes: the so-called Full-Function Devices (FFD) and the Reduced-Function Devices (RFD). FFDs are enabled to perform as network ‘Coordinators’. In that case, FFDs are in charge of the communications of a set (or “cluster”) of nodes (the “children” nodes) following a star topology. On the other hand the role of RFD (which is reserved for very simple devices with limited resources) just permits the communication (as ‘end’ nodes) with just one FFD acting as its Coordinator.

Besides, the MAC layer of IEEE 802.15.4 enables two alternative operational modes:

1. Under the non beacon-enabled or point-to-point mode, the access control is governed by non-slotted CSMA/CA (Carrier Sense Multiple Access/Collision Avoidance). According to this medium access protocol, nodes have to sense the radio medium before starting any transmission. If the channel is busy, the transmitting device has to wait a random time (set in terms of a number of “backoff” periods) before listening to the radio again. Otherwise, if the channel is idle, the device can transmit the packet and will have to wait for an acknowledgment message from the reception point. If the acknowledgment is not received in a predetermined period the node will proceed to retransmit the packet (up to a maximum number of attempts). Similarly, if two nodes begin their transmission simultaneously or a transmitting node is unaware that the radio medium in the receptor is busy, a packet collision will occur. Collisions strongly degrade the performance of CSMA algorithm as they prevent the packets to be properly received so that they have to be retransmitted (inducing delay or even data losses if retransmissions fail after applying the typical backoff algorithm of CSMA).
2. Under the beacon enabled mode, the Coordinator node periodically broadcasts a special frame (called beacon). Beacons announce the presence of the Coordinator (identifying the corresponding WPAN) and permit the synchronization of the children nodes, so the Coordinator has to broadcast a special frame (a beacon) periodically. The time between two consecutive beacons of a Coordinator is called the Beacon Interval (BI). The BI, which can vary from 15 ms to 252 s, defines an interval or superframe (whose duration can be lower than BI) which includes a Contention Access Period or CAP and a Contention Free Period (CFP). During the CAP the communications between a Coordinator and its children nodes are governed by slotted CSMA/CA. On the contrary, during the CFP time slots can be directly reserved to particular children nodes to guarantee the quality of service of time sensitive applications.

Beacon mode is recommendable when the ZigBee Coordinator (or the intermediate routers in a multi-hop 802.15.4/ZigBee cluster-tree) are powered by batteries. In that case, children nodes are obliged to wake-up just in time for receiving the beacon from their Coordinator. The beacon informs the children if they have any pending packets for them. If this is not the case and a child in turn has not data to send (or after sending its data), it can return to the sleep mode. Once the communications with the children are completed (*i.e.*, after the superframe is elapsed), the Coordinator itself can also enter into the sleep mode. This enables an important power saving in the Coordinator.

Conversely, non-beacon mode typically suits basic applications which can be deployed by a simple star topology formed by a set of wireless sensors and a Coordinator powered from the main source. In that case, the Coordinator can maintain its radio receiver on all the time so it can communicate with any sensor in any moment. The permanent activity of the Coordinator allows clients to be inactive (in the sleep mode) for unlimited intervals of time, enabling them to save energy. Thus, the sensor motes can wake up at their will (on a periodical or event-driven basis) just to transmit the sensed data or to poll the Coordinator to check if they have to receive any message.

At present most commercial 802.15.4/ZigBee motes do not support beacon mode, so in this paper, the empirical characterization and the analytical model of battery consumption will be focused on beaconless networks.

## 3. Related Work

Presently 802.15.4/ZigBee is a key technology for the development of Wireless Sensor Networks (WSNs). Consequently, during the last three years, many theoretical, simulation-based and empirical studies have been devoted to model and characterize the performance of this specification from different perspectives.

Initial scientific works [5,6] on 802.15.4 performance were essentially based on simulations. In [7–9] the performance of actual 802.15.4 communications was evaluated and compared with existing simulation models in tools such as Simulink or NS-2. The experimental testbeds presented in [8–9] also analyzed the impact of the coexistence of 802.15.4 with other wireless technologies (802.11 and/or Bluetooth) operating in the same 2.4 GHz ISM band. Results seem to indicate that 802.15.4 throughput may be seriously affected by such interferences.

In [10] authors compared non-beacon and beacon transmission modes in a realistic scenario with two IEEE 802.15.4 development boards through different performance metrics. The study concluded that beaconless networks perform better. The optimization of the association phase of the nodes in 802.15.4 networks is considered in [11]. The study in [12] briefly summarizes the current consumption of commercial chipsets of diverse standards for wireless communications, including Bluetooth, Ultrawideband (UWB), 802.11 (Wi-Fi) and 802.15.4/ZigBee technologies, during packet transmission and reception. The study reveals that 802.15.4 technology approximately requires one tenth of the current required by UWB and Wi-Fi, and less than the half intensity needed by Bluetooth chipset.

The behavior of 802.15.4 MAC, and especially the performance of CSMA/CA algorithm, has been analytical modeled in different papers such as [13–17] for beacon-enabled and/or beaconless 802.15.4 networks. The accuracy of all these models, normally based on two-dimensional Markov chains, is evaluated by simulations (normally performed with NS-2 simulator). In [18], the proposed model for slotted (beaconed) 802.15.4 MAC is employed to predict the energy consumption per received data bit as a function of the traffic load, the packet size, the initial configuration parameters of the CSMA/CA algorithm and the number of nodes in the network. However, the employed consumption model for the different states in the nodes is not justified. A similar study, also for beacon enabled cluster-trees, is presented in [19]. The study offers a mathematical formulation to compute the consumption of the ZigBee Coordinator and the end devices of the cluster-tree depending on the emitted traffic and the beacon timing. For the calculus of the power consumption the model (which assumes that the radio state is idle during the CSMA/CA backoff time) utilizes the values offered by the datasheets of Chipcon (now acquired by Texas Instruments) CC2420 radio transceiver and the Microchip PIC18LF8720 low-power microcontroller. The model is validated with a specific simulating tool for WSNs (WISENES) developed by the authors. The consumption in beaconed networks is also characterized in [20]. In that interesting paper authors present their own measurements of the power consumption of a CC2420 transceiver (although the experimental testbed for the measurements is not described). The paper also empirically characterizes the relationship between the received power and the bit error probability. Accordingly, the proposed model, which takes into account the dynamics of CSMA/CA mechanism, permits to calculate the mean required energy per data bit as a function of the path losses. However the characterization of the CSMA contention period (e.g., the average delay of the contention period or the probabilities of having a channel access failure or a collision) is carried out by means of Monte Carlo simulations for particular conditions of the traffic load.

The authors in [21] propose a method to tune the contention control of slotted CSM/CA aiming at maximizing power saving and throughput. The study, which is evaluated by simulations utilizing the battery model of a commercial radio module, defines a specific metric to calibrate the battery efficiency. However the model neglects the energy consumptions that take place for specific operations of the radio module (e.g., during the turnaround time or in the backoff intervals).

An analytic model for the interferences between IEEE 802.15.4 and IEEE 802.11 is provided and validated through OPNET simulations in [22].

The study in [23] suggests using the battery state in the 802.15.4/ZigBee nodes as a metric for the AODV (Ad Hoc on Demand Distance Vector) routing algorithm typically employed in ZigBee mesh topologies. The paper in [24] investigates the effects of employing a cryptographic mechanism on the power consumption of beacon-enabled 802.15.4 networks.

Different studies have also modeled the performance of 802.15.4/ZigBee technology when applied in specific environments. The work in [25] evaluates the applicability of beaconed 802.15.4/ZigBee to industrial monitoring WSNs. The evaluation is performed by a set of systematic simulations with OMNeT++ tool. In particular, the paper analyses the effect of the parameterization (Beacon Order, Superframe Order) of the beaconing process. The mean energy consumption per transmitted byte is computed but assuming the battery model of a CC1000 radio module [26] which is not compliant with 802.15.4 standard. A similar study, also founded on simulations, is presented in [27]. The study tries to assess the influence of the configuration of MAC parameters on the behavior of beaconless IEEE 802.15.4 networks under different traffic loads and levels of interference.

Biosensors are expected to be a major application field for 802.15.4/ZigBee. The viability of 802.15.4 technology for medical Personal Area Networks of sensors is investigated in works such as [28–30]. In particular [28] presents an analytical model to compute the lifetime of a hypothetic network of implanted 802.15.4 sensors. The study, which utilizes the typical consumption of a CC2420 chip, is carried out for both beacon and beaconless modes concluding that beaconed networks present more restrictions in term of available data rate and crystal tolerance. On the other hand, the evaluation of the capabilities of 802.15.2 communications for wireless medical applications is performed in [29] and [30] through systematic simulations with OPNET and OMNeT++. To compute the energy required per message [30] employs the consumption model documented in the datasheets of Jennic JN5139 ZigBee modules.

In this paper we intend to integrate an empirical characterization of the current consumption of actual 802.15.4/ZigBee motes under different operations and the analytical performance models of 802.15.4. The goal is to provide a simple model that permits to predict the maximum, minimum and average battery lifetime of an 802.15.4/ZigBee mote under different circumstances of traffic load, data rate and probability of packet loss.

## 4. Experimental Testbed

### 4.1. Hardware for the 802.15.4 Motes

An 802.15.4/ZigBee star, consisting of a Coordinator and a child end device (acting as the sensor mote) was created by using the eZ430-RF2480 Kit by Texas Instruments [31]. This kit includes three devices which can alternatively perform as ZigBee Coordinator, router or end device. The hardware architecture of these devices is based on the interaction between an MSP430 ultra low power microcontroller and the CC2480 2.4 GHz ZigBee processor. In addition, the board of each device incorporates a light sensor, a push-button, two LEDs, an SMD chip antenna and a connector for the external voltage supply (of up to 3.6 V) as well as 5 GPIO lines for expanding the I/O interface. One of the sensors also provides USB connectivity with a dongle interface.

The CC2480 processor, which operates in the 2.4 GHz band, belongs to the CC24XX & CC25XX family of IEEE 802.15.4/ZigBee-compliant processors. This family utilizes the Z-Stack of Texas Instruments, which is one of the most widely employed implementations of the 802.15.4/ZigBee stack for the deployment of wireless sensor networks.

In contrast with other chips that only implement an 802.15.4 transceiver, the CC2480 processor provides full ZigBee functionality, as far as it integrates the whole Z-Stack in a single chip. This permits the CC2480 to release the external microcontroller from handling all the timing critical and processing intensive protocol operations. Thus the computing power of the MSP430 can focus on the particular needs of the application. In our testbed the application was a very simple sensing application provided with the demonstration kit. The example application is designed for temperature and battery voltage sensor reporting.

### 4.2. Measurement Testbed

**Figure 1.**
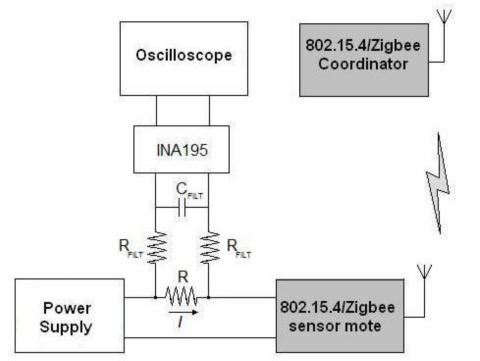
Experimental setup for the measurements of battery consumption.

The employed testbed for the measurements is sketched in Figure 1. The general idea is to monitor the power required by a sensor mote, acting as an 802.15.4 child node, when communicating with a FFD mote acting as the network Coordinator. The role of each device is decided at the beginning of each experiment by pressing (or not) a push-button during an initial configuration interval of 3 s.

In order to estimate the current drained by the sensor mote we place a shunt resistor with a known value between the voltage source (in this case a PS2520G Tektronik equipment) and the corresponding supply pin of the mote.

To minimize the voltage loss in the supply line of the mote, the shunt resistance is set to a very low value (1 Ω). As the current to measure is in the order of mA the voltage across the shunt resistor has to be amplified so it can be properly visualized in an oscilloscope (in this case, a TDS 3012B model by Tektronix). For this purpose we include an INA195 amplifier, especially conceived for current shunt monitoring. The measurement circuit also incorporates a low-pass filter formed by a capacitor (*C_FILT_*) and two resistors (*R_FILT_*). The goal is to remove high-frequency components that cannot be properly represented in the oscilloscope. To benefit from the low output impedance of the amplifier, the filter is placed at the input pins of the INA195.

To design the value of *R_FILT_*, we have to take into account that a high value can reduce the gain of the amplifier [32]:

(1) $$G_{\mathrm{INA}195}=100\cdot \frac{R_{\mathrm{INA}195}}{R_{\mathrm{INA}195}+R_{\mathrm{RFILT}}}$$

where *R_INA195_* is an internal resistance of 5 kΩ in the INA195.

On the other hand, *R_FILT_* has to be much higher than the shunt resistor *R*, so that the voltage drop in *R* is not affected by the inclusion of the filter. Considering these two restrictions we choose a *R_FILT_* of 47 Ω:

(2) $$R=1\Omega \ll R_{\mathrm{RFILT}}=47\Omega \ll R_{\mathrm{INA}195}=5k\Omega$$

Once *R_FILT_* is known, the value of the capacitor *C_FILT_* is directly derived from the desired cutoff frequency of the filter:

(3) $$f_{c}=\frac{1}{2\pi \cdot (2R_{\mathrm{FILT}})\cdot C_{\mathrm{FILT}}}$$

According to the Nyquist theorem the sampling frequency of the oscilloscope (*f_s_*) must be at least twice the value of the maximum frequency of the signal to be digitized. The horizontal resolution of the utilized oscilloscope (that is to say, the number of points across the oscilloscope display) is 10,000 samples (1,000 samples per horizontal division). Since the minimum time window that we intend to observe is 200 ms (20 ms per division), we have that the maximum sampling rate turns out to be:

(4) $$f_{s}=\frac{10000\;\mathrm{samples}}{200\;\mathrm{ms}}=50\;\mathrm{kHz}\;(\mathrm{ksamples}\;/\;s)$$

By selecting a value of 220 nF for *C_FILT_*, and applying equation (3) we accomplish that:

(5) $$f_{c}=7.7\;\mathrm{kHz}<\frac{f_{s}}{2}=25\mathrm{kHz}$$

Finally the measurement system was calibrated against a multimeter. For this purpose the ZigBee mote was substituted by a potentiometer and a calibration table was computed for different reference currents.

## 5. Measurement Results and Discussion

In the following subsections we present the obtained measurements of the drained current from the supply for the typical operations that a sensor mote performs to connect and transmit data to the 802.15.4/ZigBee Coordinator.

### 5.1. Consumption during Start-up

**Figure 2.**
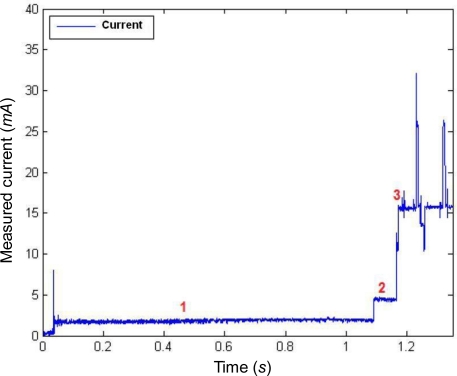
Measured supply current during the start-up of the mote.

The Figure 2 represents the instantaneous current consumption after the mote is turned on. Initially, the ZigBee processor is deactivated. Thus the consumption is solely due to the microprocessor, which operates at 8 MHz for this application. The first interval (marked as 1 in the figure) is the time imposed by the microprocessor (about 1 s) to assume that the supply voltage is stabilized. The required current is 1.8 mA approximately. During the interval no. 2, of about 0.1 s, the microprocessor calibrates the system clock with a consumption of 4.6 mA. After this calibration the MSP430 activates (interval 3) the ZigBee processor which increases the drained current to 15.4 mA. Then, the architecture remains in this state and waits until the user press the button in the board to start the wireless communications.

### 5.2. Consumption during the Association to the Coordinator

**Figure 3.**
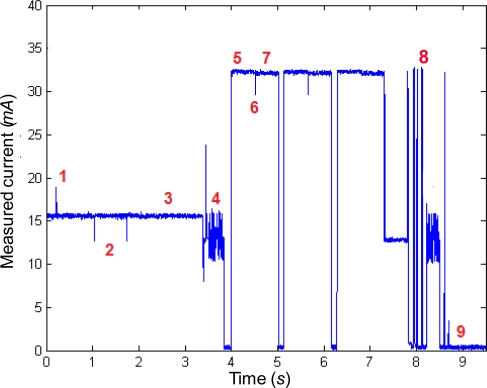
Measured supply current during the association to the Coordinator.

Before transmitting any data, the sensor has to detect the presence of the Coordinator and proceed to its association. The measured evolution of the required current during this operation is illustrated in Figure 3 and is composed of the following phases:

Intervals 1 to 3: After pressing the button to trigger the activity of the mote, the microcontroller waits for a period of about 2–3 seconds. If the button is pressed for a second time during this period (which is the case), the mote assumes to be a final device. The consumption of this phase (of 15.5 mA) is due to the fact that both the ZigBee processor and the microcontroller are simultaneously activated.

Interval 4: After the role of the mote (final device) is decided, the microcontroller sends the corresponding orders to the ZigBee processor to initiate the search for a Coordinator. After that, the microcontroller enters sleep mode (with an extremely low consumption).

Intervals 5 to 7: In non-beacon enabled networks the ZigBee Coordinator does not indicate its existence to other devices by transmitting beacon frames. Consequently the sensor motes must execute an active scanning procedure to detect the presence of the Coordinator. An active channel scan is performed over a pre-specified set of radio channels. For each channel, the mote sends a special command requesting a beacon. Upon receiving this request, any Coordinator operating in that channel in a non-beacon enabled mode will respond with a single beacon frame executing CSMA/CA. Meanwhile the mote enables its radio receiver for a predetermined time. During this time, the device rejects all non beacon frames and stores the information of all these single beacons received from the existing Coordinators. Once this listening time is over, the mote repeats the scanning operation in the following channel. In the case of the performed experiment, two different channels are explored during phases 5 and 7, respectively. The peak marked by point 6 in the figure indicates the moment of the commutation between the channels. As it can be seen from the figure the process is iterated three times. In case of receiving several single beacons the mote can choose which Coordinator to join. This election can be previously defined by upper layers. Otherwise it can be dynamically decided based on the measured peak energy in each requested channel. From the figures we can observe that in the analyzed motes the active scanning of a channel lasts for about 0.5 s, while it demands a current of 32.5 mA. This consumption is mainly due to the need of listening to the channel during a certain interval in the search of the Coordinator beacons.

Interval 8: After the scanning phase and having selected a Coordinator, an association phase is required. The unassociated device triggers the association by sending an association request command, which will be acknowledged by the Coordinator if correctly received. Then the Coordinator has a predetermined time to decide if the device request is accepted. If so, a networking short address will be assigned to the mote. After a polling packet of the mote, this new address and a status indicating the successful association is communicated to the mote in a specific association response command, which must be acknowledged by the mote.

After the association at the routing layer, a similar process (binding) is required at the ZigBee application layer. Binding permits to create a logical link between the applications in both extremes. In our experimental testbed, the binding procedure demands the exchange of seven packets between the Coordinator and the mote.

From the figure, we can see that the association and binding procedure takes about 1 s while the required current to support this process is much lower than that needed during the scanning period.

Interval 9: After the association and binding operation with the Coordinator, the ZigBee processor comes into the sleep mode. The estimation of the drained current in this state is thoroughly described in Section 5.5.

### 5.3. Consumption during Packet Transmission

**Figure 4.**
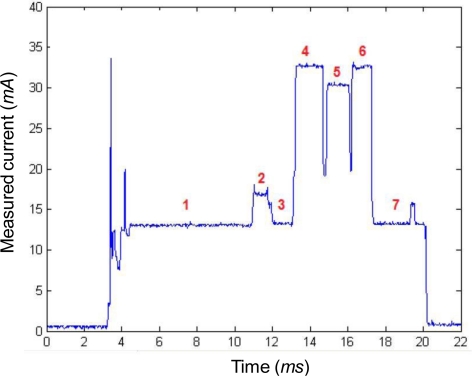
Measured supply current during the transmission of a data frame with sensed data to the Coordinator.

The evolution of the current required for the operation of transmitting the sensed data in a single packet is represented in Figure 4.

A description of the stages indicated in the figure follows:

Intervals 1, 2 and 3: this period of 10.2 ms is necessary to activate the processor of the CC2480 chip. The current demanded by the chip is 13 mA (the increase of 4 mA up to 17 mA during interval no. 2 is due to the turning-on of a red LED).

Interval 4: The radio transceiver is switched on. The current consumption rises to 32.5 mA as the module is listening to the radio channel to check whether it is idle or not. The time required by the application of CSMA/CA algorithm to get access to the radio medium obviously depends on the channel occupation. As no other sensor mote is present in this experimental testbed, the channel can be considered to be free. Thus the mean estimated duration of this phase for different iterations of this measurement (about 1.6 ms) can be regarded as the mean expected waiting time for the optimum case.

Interval 5: During this period, once the channel is assessed to be free, the radio transceiver sends to the Coordinator the sensed data (2 bytes in this case, consisting of the estimated values of the supply voltage and the temperature). Again, the experiments reflect the optimal case in which no radio collision occurs. The required current is estimated to be 30.5 mA. As characterized in Section 6, the duration of this phase depends on the size of the data to be transmitted.

Interval 6: In this phase of about 1.3 ms, the mote switches again the radio transceiver to the listening mode in order to receive the corresponding acknowledgement (ACK message) from the Coordinator. The transmission of the 11-byte ACK packet does not require applying the CSMA/CA algorithm as far as 802. 15.4 sets up a constant ‘quiet time’ after the transmission of any frame during which no node can transmit except that having to acknowledge the received packet. The consumption, as in interval 4, is 32.5 mA. After this phase the radio transceiver is deactivated.

Interval 7: This time of 9 ms is required by the CC2480 processor to send the data to the MSP430 microcontroller (which is briefly activated) and to commute to the sleep mode. As in phases 1 and 3, the activity of this processor absorbs a current of about 13 mA (during the short interval of less than 0.1 ms in which the microcontroller is activated the consumption rises to 15.4 mA).

### 5.4. Consumption due to Loss of Connection

If a sensor mote does not receive any acknowledgment from the Coordinator after resending the sensed data for a predetermined number of attempts, the ZigBee stack may assume that the device is disconnected (“orphaned”). In that case, the higher protocol layers may either reset the device (to repeat the association procedure later) or send a special realignment message indicating that the mote is orphaned. This last procedure is implemented by the utilized motes. Thus, after concluding that the connection with the Coordinator is lost, the sensor mote transmits an orphan notification command. As for the active scan before the association, this command is emitted for every channel over a pre-specified set of logical channels.

If a Coordinator, which keeps a record with its neighbors’ information, receives this orphan notification, it checks if the device was previously associated. If so, the Coordinator responds with a realignment command to the orphaned device. Otherwise, it ignores the packet. The procedure finishes when the device receives this realignment command or when the specified set of logical channels has been scanned a predetermined number of times. If the orphan scan fails, the node will assume that the association is lost and will trigger the active scan and the association phase described in Section 5.2.

**Figure 5.**
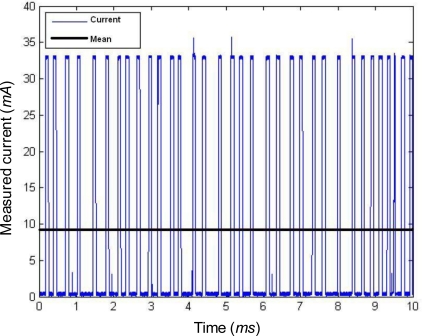
Drained current during orphan scan.

Regarding the consumption due to a permanent loss of connection, the required current depends on the number of scanned channels. Figure 5 shows the drained current if just one channel is repeatedly scanned without receiving any answer from the Coordinator. In that case, the mean drained current is 9.3 mA. On the other hand, if the number of inspected channels is 16, the consumption increases up to 27.6 mA.

### 5.5. Consumption during Sleep-Mode

**Figure 6.**
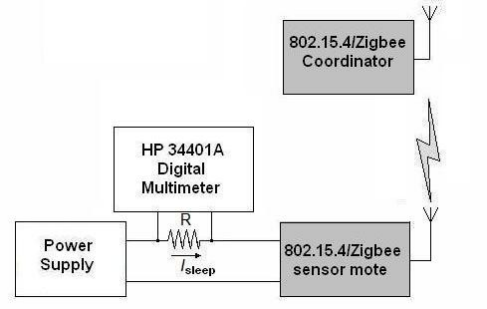
Experimental setup for the measurements of battery consumption during sleep mode.

A crucial aspect for the durability of the mote is the current consumption during the sleep mode. Since the vertical resolution of the oscilloscope and the low value of the utilized measurement resistor do not permit an accurate estimation of this current, we now use the setup depicted in the Figure 6. In this case we substitute the oscilloscope by a Hewlett Packard 34401A Digital Multimeter. When using a DC range of 100 mV this multimeter offers a precision of 3.0 μV while its input impedance is 10 MΩ. For a better estimation of the current, we increase the value of the measurement resistor R from 1 Ω to 32 Ω. For the utilized external power supply of 3.6 V, this value of 32 Ω only provokes a fall of 1.28 V in the voltage provided to the mote for a current supply of 40 mA. Consequently the minimum supply voltage (2.2 V) that the vendor specifies for the mote to operate correctly is guaranteed even when the mote demands the maximum current (about 35 mA when the radio transceiver is listening).

With this new testbed, the measured constant voltage in the resistor was estimated to be 24 μV, which indicates that current absorbed by the mote is about 750 nA. The measurements for all the described operations of the mote have been tabulated in the Table 1.

Table 2 compares these results with those reported in the datasheets of different commercial motes of the major vendors of ZigBee technology.

We repeated the previous measurements using the Texas Instruments CC2520 [35] transceiver and the Freescale MC1322x platform. Conversely to the CC2480 ZigBee processor, the CC2520 transceiver only supports the lower layers of the 802.15.4 specifications. Consequently the rest of the stack has to be implemented in a microcontroller unit. For this purpose the transceiver (in particular the EMKCC2520 evaluation model kit) was connected to the EXP430FS430 experimental board, which includes a MSP430FS430 microcontroller. On the other hand the MC1322x platform incorporates a 32-bit ARM7 microcontroller, implementing the BeeStack ZigBee protocol stack, and a compliant 802.15.4 transceiver. For the testbed we utilized the Freescale 1322x Developer Starter Kit consisting in a 1322x-SRB (Sensor Reference Board) and a 1322x-NCB (Network Coordinator Board). Both boards include a ZigBee MC1322x platform. For the measurements we isolated the consumption of the ZigBee platform from that of the rest of the elements (sensors, LEDs, *etc*.) in the experimental board.

Tables 3 and 4 summarize the obtained measurement results with this new motes, which reveal a similar consumption to that measured for the CC2480 module. In the case of MC1322x motes the main difference lies in the fact that, during the CSMA waits, the transceiver seems to remain in an idle state that permits the consumption to be reduced (this characteristic is set by default in the motes but it can be modified).

## 6. Estimation of the Battery Lifetime

In this section, taking into account the previous performed characterization of the consumed current (by CC2480 motes) during the data transmission, we propose a simple model to predict the lifetime of the battery of a sensor mote that periodically transmits information to the Coordinator.

### 6.1. Best case: Maximum Battery Lifetime

In the previous experimental testbed, the transmitted data that were periodically transmitted by the mote just consisted of 2 bytes (one to encode the sensed temperature and another one to codify the measured supply voltage). Accordingly, the time required for the data transmission (interval number 5 in Figure 4) was very low, in the same order as that needed to get access to the channel or to receive the acknowledgment from the Coordinator (time intervals 4 and 6 in the same figure). Although most typical sensed variables can be described with a reduced number of data bytes, for a generic scenario of a sensor network, sensed data may require longer payloads in the frames emitted by the 802.15.4 algorithm.

Once that the radio channel is detected to be idle (after the random waiting time imposed by CSMA/CA algorithm), the time *t_tx_(n)* that is required to transmit a frame with a data payload of *n* bytes can be computed as:

(6) $$t_{\mathrm{tx}}\;(n)=\frac{8\cdot (O_{\mathrm{MAC}}+n)}{r}$$

where *r* is the binary rate of 802.15.4, 250 kbps when operating at ISM 2.4 GHz band (Note: *The 2006 revision of the standard allows different modulations when the node works in the 868/915 MHz ISM bands. These new modulations permit to improve the bit rate up to 100 Kbps* (*for the 868 MHz band*) *and 250 Kbps* (*for the 915 MHz band*)*. Conversely, in 802.15.4 devices operating in 2.4 GHz ISM band*, *the only permitted instantaneous bit rate is 250 kbps as long as just QPSK modulation*, *with 2 Megachip/s and 62.5 Ksymbol/s*, *is enabled*). *O_MAC_* is 31 bytes, corresponding to the total overhead (preamble, frame delimiter, MAC header and CRC field) of an 802.15.4 frame.

Using *t_tx_(n)* and based on the measured values of the previous section (for the CC240 motes), we can estimate the mean current that must be supplied to the mote to activate it so it can transmit a packet of *n* bytes flowing from the application layer:

(7) $$I_{\mathrm{active}}\;(n)=\frac{t_{\mathrm{onoff}}\;I_{\mathrm{onoff}}+t_{\mathrm{listening}}\;I_{\mathrm{listening}}+t_{\mathrm{tx}}\;(n)I_{\mathrm{tx}}}{t_{\mathrm{act}}\;(n)}$$

where *t_onoff_* and *I_onoff_* are the total time (13 ms) and current (13 mA) necessary to wake up and turn off the transceiver as well as to transmit the data to the microcontroller, *t_listening_* (2.9 ms) and *I_listening_* (32.5 mA) are in turn the time and current required to access the radio channel (using CSMA/CA) and to receive the corresponding ACK from the Coordinator. Similarly *I_tx_* represents the 30.5 mA absorbed by the mote during the transmission of the *n* bytes. Finally, *t_act_(n)* indicates the time of the complete activity period:

(8) $$t_{\mathrm{act}}\;(n)=t_{\mathrm{onoff}}+t_{\mathrm{listening}}+t_{\mathrm{tx}}\;(n)$$

If *T* is the update period of the data (*i.e.*, the time between two consecutive transmissions of the sensed magnitudes) we have that the mean current at which the battery is drained is:

(9) $$I_{\mathrm{drain}}\;(n)=\frac{t_{\mathrm{act}}\;(n)}{T}\;I_{\mathrm{active}}\;(n)+\left(1-\frac{t_{\mathrm{act}}\;(n)}{T}\right)\cdot I_{\mathrm{sleep}}$$

where *I_sleep_* is the current in the sleep mode while the term $\left(\frac{t_{\mathrm{act}}\;(n)}{T}\right)$ actually represents the duty cycle of the sensor mote.

Neglecting the current required for the start-up phase as well as assuming that no polling takes places so that the only existing data traffic is upstream (*i.e.*, from the mote to the Coordinator), the lifetime *L* (in years) of a supply battery of capacity *C* (expressed in mAh) can be directly derived from *I_drain_*:

(10) $$L=\frac{C\;/\;(365\cdot 24)}{I_{\mathrm{drain}}\;(n)}$$

**Figure 7.**
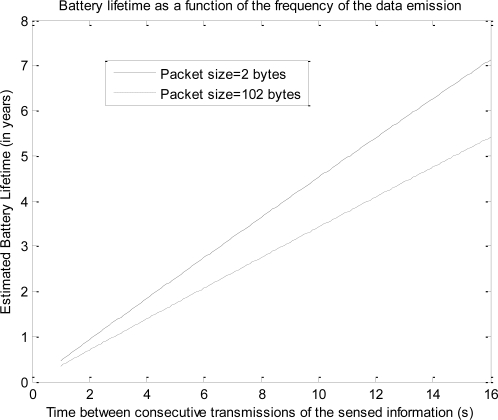
Expected Battery lifetime (in optimal conditions) of a mote as a function of the frequency of data emission for two different sizes of sensed data (battery capacity is assumed to be 1,200 mAh).

**Figure 8.**
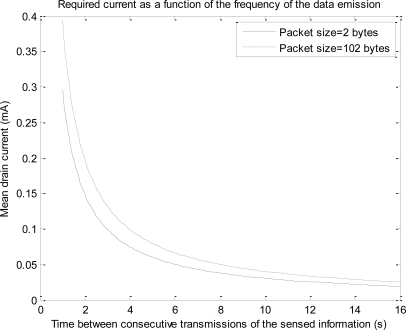
Expected mean drain current (in optimal conditions) of a mote as a function of the frequency of data emission for two different sizes of sensed data (battery capacity is assumed to be 1,200 mAh).

Figure 7 and Figure 8 depict the estimation of *I_drain_* and *L* for different values of the periodicity at which the sensed data are transmitted, ranging from 0.1 to 16 s. The curves consider a typical capacity of 1,200 mAh for the two AAA batteries that can power the mote. The figures include two extreme cases for the value of the size of the data (*n*): 2 bytes (as in the case of the experimental tests) and 102 bytes (which is the maximum admissible value of the 802.15.4 MAC payload). Most sensed magnitudes in practical sensor networking applications can be represented by a number of bytes between these two extremes (normally some bytes are enough), so the figures show that 802.15.4/ZigBee technology provides a typical maximum battery lifetime of up to several years for many typical scenarios of mote networks.

In the experimental testbed from which we obtained the estimated values of the drained currents, just one mote was interacting with the Coordinator. Similarly, the interferences of other transmitting devices (e.g., Bluetooth or Wi-Fi) in the 2.4 GHz band did not prove to have any practical influence on the availability of the radio channel (the Coordinator was placed at less than 50 cm from the sensor mote). Therefore no collisions were detected and the delay introduced by CSMA/CA backoff algorithm can be considered to be minimized. Consequently, this estimation of the lifetime (*L*) can be regarded as a maximum bound as long as in the modeling of the communications at the MAC layer, we also assume the optimal case in which no frame retransmission takes place. In the following sub-sections we extend the model for battery consumption (redefining the values of the times *t_listening_* and *t_tx_(n)*) to characterize the impact of the packet retransmissions and node re-association that may take place when channel access failures occur.

### 6.2. Worst Case: Minimum Battery Lifetime (without Re-Association)

According to the IEEE 802.15.4 MAC specification, nodes desiring to transmit a packet compete for the transmission channel following the CSMA/CA algorithm. Thus, initially, the source node must wait a certain number of slots or backoff periods (of 20 symbols, or 0.32 ms when operating in the 2.4 GHz band with 62.5 Ksymbols/s). This number is randomly selected between 0 and (2*^BE^*−1), being *BE* the backoff exponent, an increasing variable that governs the CSMA waiting times. After this random time, the node performs a CCA (Clear Channel Assessment) to check the availability of the radio channel. If the channel is busy, *BE* is incremented by 1 (up to a maximum) and the procedure is repeated. If the CCA does not succeed after a certain number of CSMA waits, the node considers that a channel access failure has occurred and the packet transmission is discarded. On the other hand, if the channel is found to be available in any CCA operation, the radio transceiver of the node changes from the reception mode to the transmission mode (as the 802.15.4 communications are half-duplex) and the packet is transmitted. The transmission is only considered to be successful if an acknowledgment packet (ACK) from the target node is received before a predetermined interval. This ACK packet is sent by the destination node as soon as the data packet arrives (CSMA/CA algorithm is not applied for the packet acknowledgements). However, the transmitted packet or its corresponding acknowledgment may experience a collision due to the activity of neighbor 802.15.4 nodes or to the interference of other devices operating in the same 2.4 GHz band. In that case, the acknowledgment will not be received, which will cause the source node to repeat the whole CSMA/CA process (resetting the *BE* variable to its initial value). The number of transmission attempts is also limited by the specification. Therefore, when this maximum number of retransmissions is reached without acknowledging, the MAC layer assumes a sending failure and the transmission is cancelled.

Consequently, the maximum time (and the maximum battery consumption) required for a successful transmission takes places when the packet is correctly acknowledged only in the last admitted transmission attempt and after iterating (for each attempt) the CCA operation the maximum number of times with the maximum delay provoked by the successive random CSMA waits.

Analytically, for this worst case, the listening time of the radio transceiver can be computed as:

(11) $$t_{\mathrm{listening}}=(\mathrm{aMaxF}+1)(t_{\mathrm{CSMA}(\text{max})}+t_{\mathrm{TA}}+t_{\mathrm{ACK}})$$

where *aMaxF* is the maximum number of times that a transmission can be retried (in the standard this parameter, *macMaxFrameRetries*, is set to 3 by default), *t_TA_* is the turnaround time (0.192 ms or 12 symbols), reserved for the transceiver to switch from reception to transmission (or vice versa) while *t_ACK_* is the maximum time (0.864 ms or 54 symbols) that the receiver waits for the ACK before proceeding with the next attempt. The term *t_CSMA(max)_* describes the maximum delay that the CSMA/CA algorithm and the CCA operations may introduce in each transmission attempt. From the previous analysis of the consumption in the utilized 802.15.4 CC2480 motes we have detected that, during the CSMA waits, the radio transceiver stays in the receiving mode. Thus, we have included a delay of the CSMA in the computation of the listening period. The term *t_CSMA(max)_* can be calculated as:

(12) $$t_{\mathrm{CSMA}(\text{max})}=\sum_{i=0}^{\mathrm{mMaxb}}\left((2^{\text{min}(\mathrm{macMinBE}+i,\;\mathrm{macMaxBE})}-1)\cdot t_{\mathrm{backoff}}+t_{\mathrm{CCA}}\right)$$

where *mMaxb* is the maximum number of times that the CSMA algorithm is repeated after the first CCA failure. According to the standard, the default value for this parameter (called *macMaxCSMAbackoffs*) is 4. The terms *macMinBE* (3 by default) and *macMaxBE* (5 by default) are the initial and maximum values of the backoff exponent (*BE)* respectively, *t_CCA_* is the time necessary for a CCA operation (8 symbols or 0.128 ms), while *t_backoff_* is the duration of a backoff period (20 symbols or 0.32 ms).

Assuming the default values for all the constants, we have that *t_CSMA(max)_* and *t_listening_* are 37.44 ms (2,340 symbols) and 153.98 ms (9,624 symbols), respectively. Similarly, the calculus of the maximum time that the radio transceiver may stay in the transmission mode has now to take into consideration that the packet can be retransmitted up to *aMaxF* times:

(13) $$t_{\mathrm{tx}}\;(n)=(\mathrm{aMaxF}+1)\cdot \left(\frac{8\cdot (O_{\mathrm{MAC}}+n)}{r}\right)$$

### 6.3. Average Case: Mean Battery Lifetime (without Re-Association)

The mean expected battery consumption depends on the frequency of the collisions and the channel access failures. If we assume that both processes (access failures and collisions) follow independent and self-uncorrelated stochastic processes, the previous equations for the worst case can be straightforwardly modified to define the listening and transmission periods for the average case.

In particular, if *p_o_* denotes the probability that the channel is occupied when the CCA operation is performed and *p_c_* is the probability of a packet collision, *i.e.*, the probability that a packet is not acknowledged after being transmitted, (—See [20] for an empirical characterization of the bit error probability as a function of the received power. Authors in [16] offer an analytical expression to compute the probabilities *p_o_* and *p_c_* as a function of the number of nodes contending in the 802.15.4/ZigBee network. The expression does not take into account the presence of other interfering sources—), the average listening time in the radio transceiver required to transmit a packet can be computed as:

(14) $$t_{\mathrm{listening}}=\sum_{i=0}^{\mathrm{aMaxF}}{\left(1-p_{\mathrm{CSMAfail}}\right)}^{i}\cdot p_{c}^{i}\cdot \left\{p_{\mathrm{CSMAfail}}\cdot t_{\mathrm{CSMAfail}}+(1-p_{\mathrm{CSMAfail}})\cdot (t_{\mathrm{CSMAnofail}}+t_{\mathrm{TA}}+t_{\mathrm{ACK}})\right\}$$

where:

-*p_CSMAfail_* represents the probability of suffering a channel access failure (after *mMaxb*+1 CSMA waits and *mMaxb*+1 failed CCA operations):
  (15) $$p_{\mathrm{CSMAfail}}=p_{o}^{\mathrm{mMaxb}+1}$$
-*t_CSMAfail_* describes the mean time of a transmission attempt that concludes in a channel access failure, after (*mMaxb*+1) CSMA waits and (*mMaxb*+1) CCA failures.
  (16) $$t_{\mathrm{CSMAfail}}=\sum_{i=0}^{\mathrm{mMaxb}}\left(\frac{1}{2}(2^{\text{min}(\mathrm{macMinBE}+i,\mathrm{macMaxBE})}-1)\cdot t_{\mathrm{backoff}}+t_{\mathrm{CCA}}\right)$$

With the default values defined for the specification for *mMaxb*, *macMinBE* and *macMaxBE*, the computation of the previous expression results in a value for *t_CSMAfail_* of 19.04 ms (1,190 symbols at a rate of 62.5 Ksymbols/s).

-*t_CSMAnofail_* stands for the mean expected delay introduced by the CSMA/CA algorithm and CCA operations of an attempt that does not finish in a channel access failure (that is to say, an attempt with a successful CCA). This time can be computed [16] as:
  (17) $$t_{\mathrm{CSMAnofail}}=\left(\frac{1-p_{o}}{1-p_{o}^{\mathrm{mMaxb}+1}}\right)\sum_{i=0}^{\mathrm{mMaxb}}p_{o}^{i}\left\{\sum_{j=0}^{i}\left(\frac{1}{2}(2^{\text{min}(\mathrm{macMinBE}+j,\mathrm{macMaxBE})}-1)\cdot t_{\mathrm{backoff}}+t_{\mathrm{CCA}}\right)\right\}$$

On the other hand, the mean time that the radio transceiver is in the transmission state (for a packet payload of n data bytes) is:

(18) $$t_{\mathrm{tx}}\;(n)=\left(\frac{8\cdot (O_{\mathrm{MAC}}+n)}{r}\right)\cdot \sum_{i=0}^{\mathrm{aMaxF}}{\left(1-p_{\mathrm{CSMAfail}}\right)}^{i+1}\cdot p_{c}^{i}$$

In the previous expression, the summation $\sum_{i=0}^{\mathrm{aMaxF}}{\left(1-p_{\mathrm{CSMAfail}}\right)}^{i+1}\cdot p_{c}^{i}$ is the mean number of times that a packet is transmitted.

**Figure 9.**
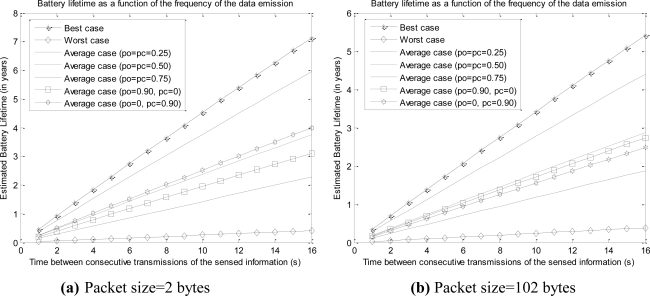
Comparison of the expected battery lifetimes for the different analyzed cases and different probabilities of CCA failure (*p_o_*) and packet collision (*p_c_*) (battery capacity is 1,200 mAh).

Figure 9 compares the battery lifetime of the different analyzed cases (assuming again a battery capacity of 1,200 mAh) for two sizes of the data payload (2 and 102 bytes). For the average case, the results have been obtained for diverse values of the probabilities p_o_ and p_c_. The graphs illustrate the strong divergences between the different contemplated situations. The worst case is shown to be excessively pessimistic as even scenarios with high values for the probabilities p_o_ and p_c_ exhibit a considerably longer average lifetime.

### 6.4. Effect of the Node Re-Association on Power Consumption

In the previous subsections, the battery consumption of the mote has been calculated considering that nodes just associate to the 802.15.4/ZigBee network once during the whole battery lifetime (just after the initial start-up). Thus the power consumption is basically due to the periodical transmission attempts and the current drained during the active scanning as well as during the association and binding phases is negligible for the estimation of the battery lifetime. However, in most cases, after a packet loss (provoked by successive collisions or by a channel access failure), the mote will be programmed to search and re-associate with a Coordinator (if the orphan scanning is not implemented or if the realignment command is not received after the orphan scan). As shown in section 5, this re-association process (which includes the active scanning as well as the exchange of messages with the Coordinator to proceed with the association and binding phases) may last for several seconds with a mean current consumption of more than 20 mA. Consequently, the packet losses may introduce an extra component that must be incorporated to the proposed model of battery consumption. In particular, the activity interval required to transmit a packet has to be increased as follows:

(19) $$t_{\mathrm{act}}\;(n)=t_{\mathrm{onoff}}+t_{\mathrm{listening}}+t_{\mathrm{tx}}\;(n)+p_{f}\cdot t_{\mathrm{reassoc}}$$

where *t_reassoc_* is the time required for the whole re-association process while *p_f_* describes the probability of packet loss. This probability can be directly computed [16] from the probability of packet collision (*p_c_*) and the probability of channel access failure (*p_CSMAfail_*), defined in equation (15), as:

(20) $$p_{f}={\left(1-p_{\mathrm{CSMAfail}}\right)}^{\mathrm{aMaxF}+1}\cdot p_{c}^{\mathrm{aMaxF}+1}+\sum_{i=0}^{\mathrm{aMaxF}}\left(p_{\mathrm{CSMAfail}}\cdot {\left(1-p_{\mathrm{CSMAfail}}\right)}^{i}\cdot p_{c}^{i}\right)$$

where *aMaxF* is the aforementioned variable that defines the maximum number of retransmission attempts.

Similarly the mean current (*I_active_*(*n*)) that is drained when the transceiver is not in sleep mode has to be redefined as:

(21) $$I_{\mathrm{active}}\;(n)=\frac{t_{\mathrm{onoff}}\;I_{\mathrm{onoff}}+t_{\mathrm{listening}}\;I_{\mathrm{listening}}+t_{\mathrm{tx}}\;(n)\;I_{\mathrm{tx}}+p_{f}\cdot t_{\mathrm{reassoc}}\;I_{\mathrm{reassoc}}}{t_{\mathrm{act}}\;(n)}$$

where *I_reassoc_* is a new term that defines the mean current required during the re-association phase.

The particular values of *I_reassoc_* and *t_reassoc_* obviously depend on the number of scanned channels and on the probability of suffering packet losses during the re-association. If we consider the most favorable case (with just one scanned channel and assuming that the re-association is always successful), the values of *I_reassoc_* and *t_reassoc_* for our analyzed motes are 2 s and 26.6 mA, respectively.

**Figure 10.**
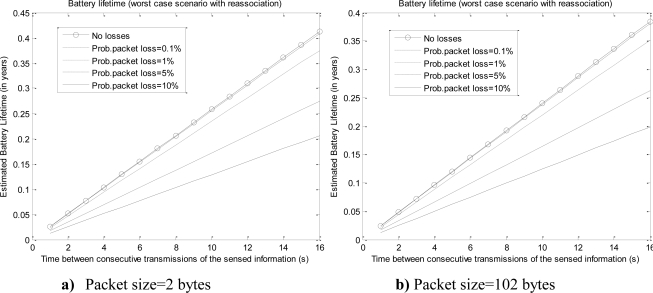
Effect of the re-association on the minimum predicted battery lifetime.

Figure 10 depicts the expected minimum lifetime for different values of the packet loss probability (0.1, 1 and 10%). The figures show that only high packet loss probabilities can seriously impact on this worst case scenario. Conversely, if we consider the average consumption (with the average values for *t_listening_* and *t_tx_(n)* calculated in Section 6.3), the effect of the re-association is more evident.

**Figure 11.**
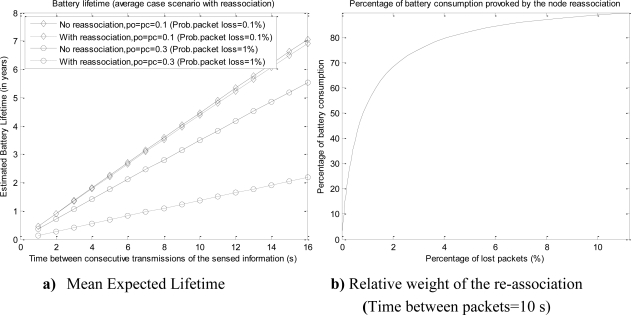
Effect of the re-association on the average battery consumption (Packet size = 2 bytes)

Figure 11(a) compares the mean battery consumption for the cases with and without re-association (after packet losses) for two different loss probabilities (the loss probability is calculated from the selected values for *p_o_* and *p_c_*). The figure (computed for a packet payload size of 2 bytes) shows that even for a low packet loss (0.1%) the current drained by the association process cannot be ignored to estimate the expected mean lifetime of the battery. Figure 11(b) confirms this idea. This figure depicts the evolution of the relative weight of the re-associations on the mean battery consumption as a function of the packet loss probability (for a packet rate of 0.1 packet per second). In order to compute this evolution, *p_o_* and *p_c_* were set to the same value and modified from 0.01 to 0.50 with increments of 0.01. The figure clearly evidences that even for packet loss probabilities lower than 2%, the battery consumption is already dominated by the re-association processes. This effect cannot be disregarded in noisy scenarios with many interfering sources or in dense 802.15.4/ZigBee networks where the collision probability is high.

## 7. Conclusions

The 802.15.4/ZigBee protocols are a promising technology in the ambit of low-power sensor networking. This paper has provided a full experimental characterization of current consumption in actual 802.15.4/ZigBee sensor motes. The characterization thoroughly takes into account the different operations required by 802.15.4 protocol, not only to transmit data, but also during the connection phase of the end device.

The empirical modeling of the consumption has prompted a practical and simple formula that permits to forecast the battery lifetime of a sensor mote as a function of the duty cycle and the size of the data to be transmitted. The proposed model defines the maximum, minimum and mean expected battery lifetime, taking into consideration the delay introduced by the CSMA/CA algorithm applied by the 802.15.4 MAC layer. The model has also been extended to cope with the extra consumption that the node re-association requires when a packet loss occurs.

The characterization presented has focused on different commercial devices that implement Z-stack, the Texas Instruments (TI) version of the ZigBee stack. Nevertheless, the study has also analyzed the current consumption of other 802.15.4/ZigBee devices (in particular one using the CC2520 transceiver, also by Texas Instruments, and the Freescale MC1322x platform). The performed battery lifetime study can be easily extended for these devices, just modifying the current drained during the different operations of the motes. Future work should repeat the characterization of the consumption with chips of other vendors implementing other popular versions of ZigBee technology such as EmberZNet PRO by Ember [39] or JenNet by Jennic [40].

## Figures and Tables

**Table 1.** Summary of drained current for different 802.15.4/ZigBee operations in the CC2480 mote.

**Operation**	**State**	**Mean Required Current (mA)**	**Duration (ms)**
Inactivity	Sleep mode	0.00075 mA	Variable
Start-up	Power-up of the microcontroller	2 mA	1,100 ms
	Waiting period (microcontroller and ZigBee processor are active)	15.5 mA	Variable
Association to the Coordinator	Scanning in 1 channel	26.6 mA	2,000
	Scanning in 16 channels	33.8 mA	up to 27,500
Transmission of a packet of *n* bytes with sensed data	Transmission of a *n-* byte packet	30.5 mA	0.99 + (8 × *n*)/250
	Listening of the channel: CSMA wait, CCA, Reception of ACK	32.5 mA	2.9 ms
	Activation/deactivation of the ZigBee processor (radio transceiver is off)	13 mA	13 ms
Loss of connection (orphan scan followed by active scan without answer)	Scanning in 1 channel	9.3 mA	Variable
	Scanning in 16 channels	27.6 mA	Variable

**Table 2.** Comparison of the performed measurements with the current consumption (at 0 dBm) reported in the datasheets of different commercial 802.15.4 compliant transceivers.

**State**	**Measurements (CC2480+MSP430)**	**TI (chipcon) CC2420** [33]	**TI CC2480** [34]	**TI CC2520** [35]	**Ember EM260** [36]	**Jennic JN5121** [37]	**Freescale MC1322x** [38]
Operating Voltage (V)	3.6 V	2.1–3.6 V	2.0–3.6 V	1.8–3.8 V	2.7–3.6 V	2.1–3.6 V	2.0–3.6 V
Output power range (dBm)	0 dBm	−24 to 0 dBm	0 dBm	Up to 5 dBm	0 dBm	−32 to 4.5 dBm	−30 to 5 dBm
Sleep mode	0.75 μA	1 μA (max)	0.5 μA	0.12 μA (max.)	3.5 μA	1 μA	0.4 μA
Transmission	30.5 mA	17.4 mA	27 mA	25.8 mA	49 mA	28–30 mA	29 mA
Channel Listening	32.5 mA	18.8 mA	27 mA	22.3 mA	44 mA	28–34 mA	21 mA

**Table 3.** Summary of drained current for different 802.15.4/ZigBee operations in the CC2520 mote.

**Operation**	**State**	**Mean Required Current (mA)**	**Duration (ms)**
Inactivity	Sleep mode	0.000030 mA (only transceiver)	Variable
Association to the Coordinator	Scanning in 1 channel	27.5 mA	0.5 s
Transmission of a packet of *n* bytes with sensed data	Transmission of a *n-* byte packet	27 mA	0.99 + (8 × *n*)/250
	Listening of the channel: CSMA wait, CCA, Reception of ACK	25.6 mA	2.1 ms
	Activation and programming of the radio transceiver	6.7 mA	1.3 ms
Loss of connection (orphan scan followed by active scan without answer)	Scanning in 1 channel	15.7 mA	Variable

**Table 4.** Summary of drained current for different 802.15.4/ZigBee operations in the MC1322x mote.

**Operation**	**State**	**Mean Required Current (mA)**	**Duration (ms)**
Inactivity	Sleep mode	0.3 μA	Variable
Association to the Coordinator	Scanning in 1 channel	15.56 mA	2.85 s
Transmission of a packet of *n* bytes with sensed data	Transmission of a *n-* byte packet	32 mA	0.99 + (8·*n*)/250
	Listening of the channel: CCA and Reception of ACK	25.5 mA	0.68 ms
	CSMA wait, Activation/deactivation of the radio transceiver	10 mA	1.2 ms
Loss of connection (orphan scan followed by active scan without answer)	Scanning in 1 channel	21.8 mA	Variable
